# Immediate Placement of an Implant Connected to a Customized Zirconia Membrane in Compromised Sockets: First Two Case Reports

**DOI:** 10.1002/ccr3.73024

**Published:** 2026-06-25

**Authors:** Laith Glissa, Latifa Berrezouga

**Affiliations:** ^1^ Private Practice, Cabinet Dr. Laith Glissa Msaken, Sousse Tunisia; ^2^ Faculty of Dental Medicine University of Monastir Monastir Tunisia; ^3^ Medical Microbiology, Immunology, Endodontics, Oral Medicine and Implantology, Faculty of Dental Medicine University of Monastir Monastir Tunisia

**Keywords:** compromised socket, digital dentistry, immediate implant placement, zirconia membrane

## Abstract

The authors described a novel technique to achieve the stability of the implant immediately placed in compromised sockets without osteotomy. Digital planning of the implant and customized zirconia membrane (CZM) design and milling were previously performed in the maxilla and the mandible. The study involved two healthy patients with alveolar bone defect. The CZM was created as a single continuous piece, and uniquely dimensioned to fit the zone of the bone defect after tooth extraction. An extension was created at the inner surface of the CZM to serve as a connector or abutment used to connect the implant to the CZM, secured with an abutment screw. As no osteotomy was performed in the two cases, the assembly CZM/Implant was stabilized with fixation screws to the cortical bone. In the first case, a crestal sinus lift was performed in the maxilla, using bovine bone graft, with immediate placement of the assembly CZM/Implant after extraction of the first molar. In the second case, immediate implantation was performed after the extraction of the second molar with a large periapical lesion. A CBCT scan confirmed the good osseointegration of the implants at 2 years. As this novel technique is promising, a research study is planned to better assess clinical outcomes and validate the new concept.

## Key Clinical Message

To improve patients' clinical outcomes, we describe the “Hanged Concept”, which is a novel technique for immediate implant placement after tooth extraction in compromised sockets. A customized zirconia membrane connected to the implant was used to achieve implant stability. The assembly was secured with fixation screws to the cortical bones.

## Graphical abstract

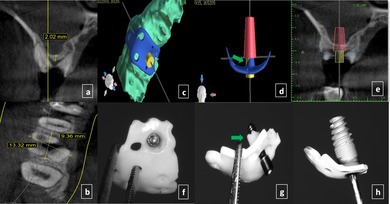

## 1 Introduction

Currently and according to the Glossary of Oral and Maxillofacial Implants, Primary stability (PS) is clinically defined as “the implant immobility at the time of surgical placement, resulting from intimate contact of the implant with the bony walls of the osteotomy. Primary stability decreases with time as osseous remodeling occurs. It is distinct from secondary implant stability, which is the result of new bone formation and osseointegration” [1, 2, 3]. Insertion torque (IT), Resonance Frequency Analysis (RFA) measured as implant stability quotient (ISQ), and X‐ray microtomography are amongst clinical methods allowing the evaluation of implant stability. A torque value ranged from 30 to 45 N cm was reported as the immediate loading threshold, and a high ISQ value (> 60) correlates with greater implant stability during osseointegration and a better clinical outcome [4]. Factors influencing implant PS are patient‐related and implant‐related factors. The quality and the quantity of bone, the implant design (shape or geometry), and implant material (titanium, ceramic, polymer) and the surgical technique were reported as influencing factors [5]. To reduce or stop crestal bone loss and improve osseointegration, autogenous bone grafts, allografts, synthetic biomaterials, and xenografts (bovine) have been implemented. As an alternative bone substitute, bovine xenograft is an indication for maxillary sinus augmentation [6, 7]. Indeed, guided bone regeneration (GBR) mesh has been used to stabilize the implant, especially in sockets with poor bone volume in immediate implant placement. In recent years, zirconia membranes (ZM) have gained attention thanks to their mechanical and biocompatibility properties.

The objective of the present case reports was to describe a novel technique of placing immediate implants, with no osteotomy preparation, in compromised sockets after tooth extraction. The technique named the “Hanged Implant Concept” or HIC doesn't rely on the commonly reported PS, but consists of the digital design of a customized zirconia membrane (CZM) connected, through a connector, to the implant. The stability of the assembly CZM/Implant is secured using osteosynthesis screws on the buccal and palatal/lingual bone plates.

## 2 Patient Information

All clinical reports (digital design and surgeries) were performed by the same operator (L.G.), an experienced oral surgeon and digital dentistry expert. The clinical project was submitted by the authors (L.G. and L.B.), according to the Declaration of Helsinki involving human subjects, to the Ethics Committee of the faculty of medicine of Monastir, Tunisia; and was approved under the following reference number IORG 0009738 N°199 OMB 0990‐0279. Informed consent was obtained from the two patients.

The selected two patients were young and healthy with no systemic or periodontal diseases or tobacco consumption history. They presented for a tooth extraction with compromised sockets (bone defect).

## 3 Clinical Findings and Diagnostic Assessment

**FIGURE 1:**
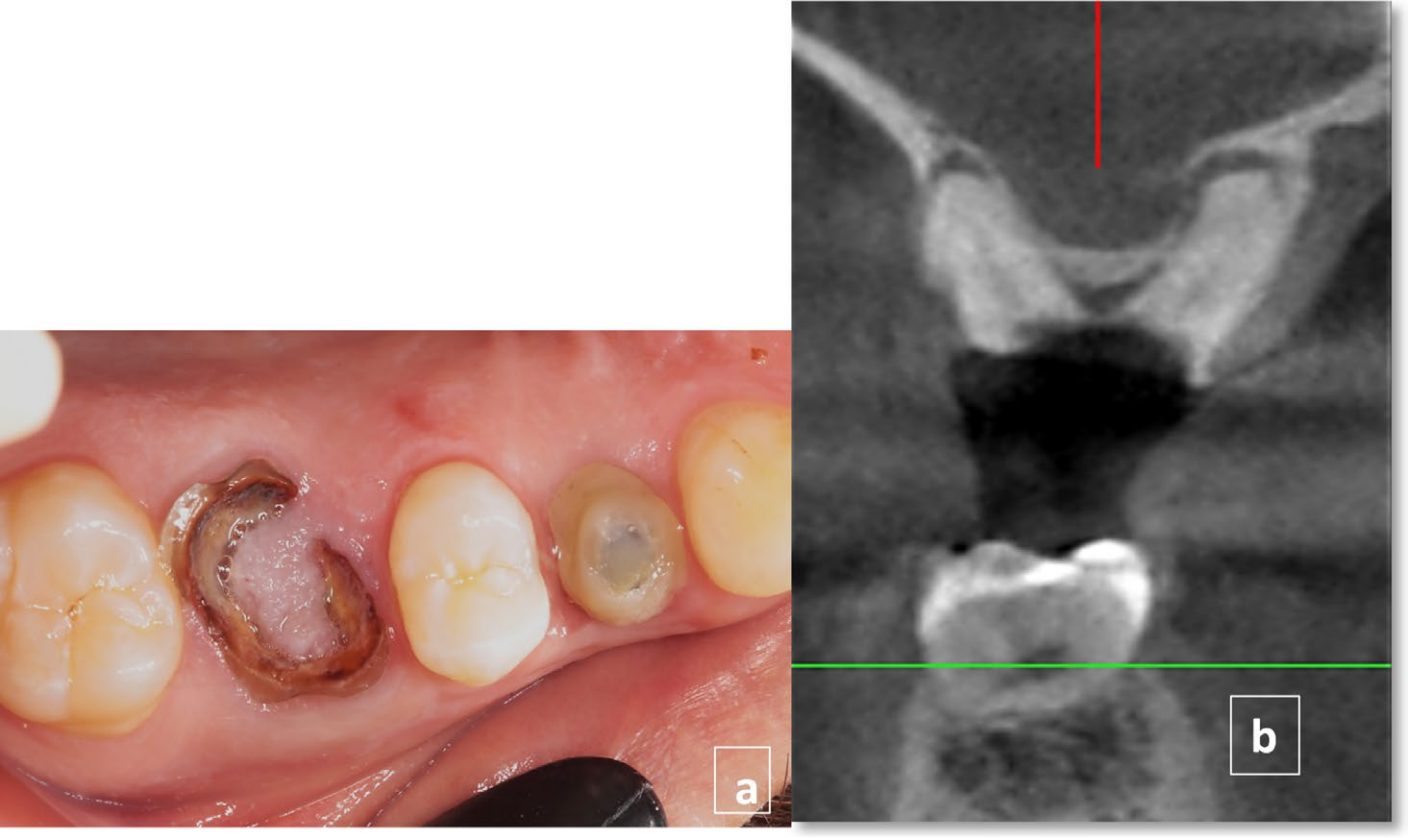
(a) Preoperative clinical view of the decayed maxillary first right molar; (b) CBCT coronal view showing periapical lesions and significant bone defect.

**FIGURE 2:**
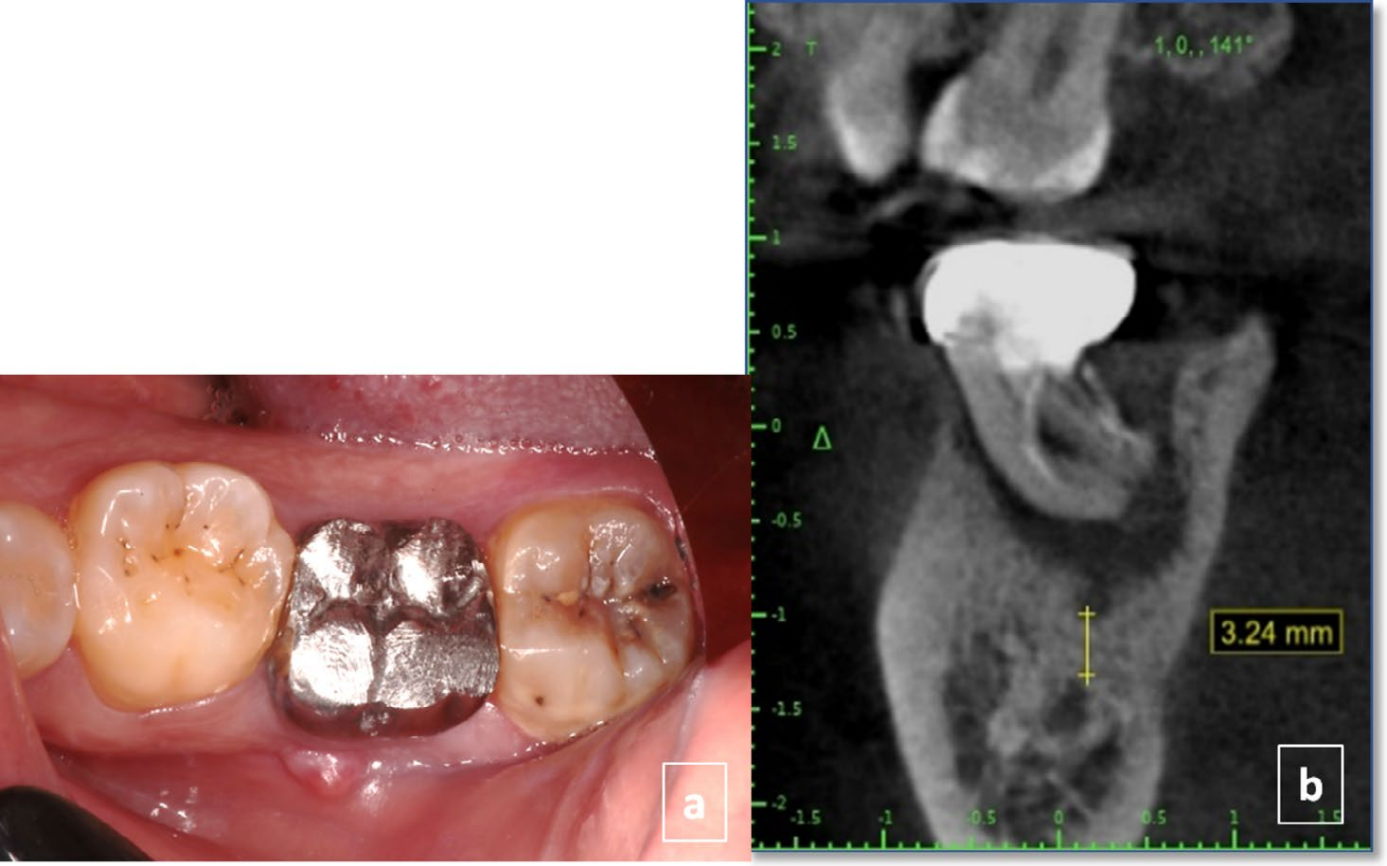
(a) Preoperative clinical view of the second mandibular right molar showing a chronic abscess; (b) CBCT coronal view of the large periapical lesion with significant alveolar bone resorption. A vertical fracture is visible on the distal root.

Clinical examination revealed healthy oral mucosa and radiographic investigation, using cone beam computed tomography (CBCT), was performed to assess bone quality and quantity, bone width and height and the relation of the concerned tooth with anatomical structures, such as the sinus in the maxilla and the alveolar nerve in the mandible. In the first case report a 30‐year‐old healthy male presented to our clinic with a severely decayed right upper first molar (tooth #16). Clinically, the tooth was asymptomatic, fractured with no remaining crown but exhibited healthy surrounding gingival tissue. A CBCT scan revealed that the roots were separated by a thin bone plate (approximately 2 mm) beneath the sinus floor. Small periapical lesions were also present (Figure 1a,b). The second case report concerned a 36‐year‐old healthy female presented with moderate pain when chewing on the second right mandibular molar (tooth #47). The tooth restored with a metallic crown was mobile (grade 3) with mild symptoms at axial percussion, and a draining fistula located at the attached gingiva indicating a chronic abscess. The periodontal probe reached the apex of the tooth (not shown). A vertical root fracture (VRF) was strongly suspected. Coronal CBCT view showed an extensive alveolar bone resorption related to a vertical fracture of the distal root (Figure 2a,b). These two unfavorable diagnoses justified the extraction of the teeth and immediate implantation.

## 4 Therapeutic Intervention

The treatment was carried out in three stages. First, the digital Workflow consisted of data acquisition and integration (IOS for intraoral scan and CBCT) and the planning of the implant position and CZM design and milling using Computer‐Aided Design and Computer‐Aided Manufacturing (CAD/CAM) technology; as well as the future prosthesis design; followed, secondly, by the surgical procedure and the immediate placement of the complex CZM/Implant and finally the CAD/CAM prosthetic restoration.

**FIGURE 3:**
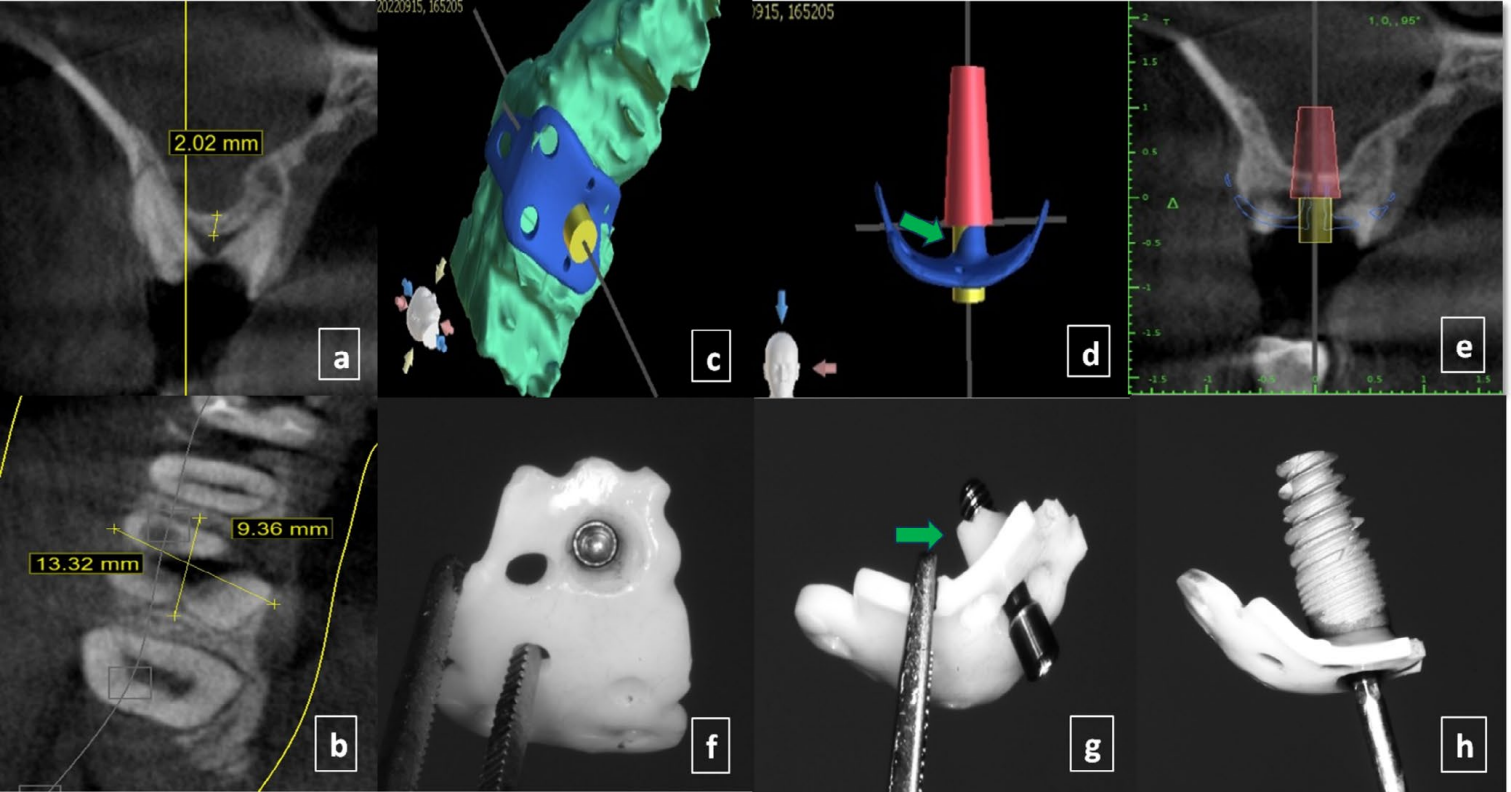
Digital planning of the implant and CZM in the maxilla. (a, b) CBCT measures; (c) ZM in blue and axis of the future abutment in yellow; (d) connection of the implant (red) to the ZM connector at the inner surface (green arrow); (e) position of the assembly ZM/Implant; (f) ZM for clinical use with perforations; (g) the ZM connector; (h) screw fixation of the implant to the ZM.

**FIGURE 4:**
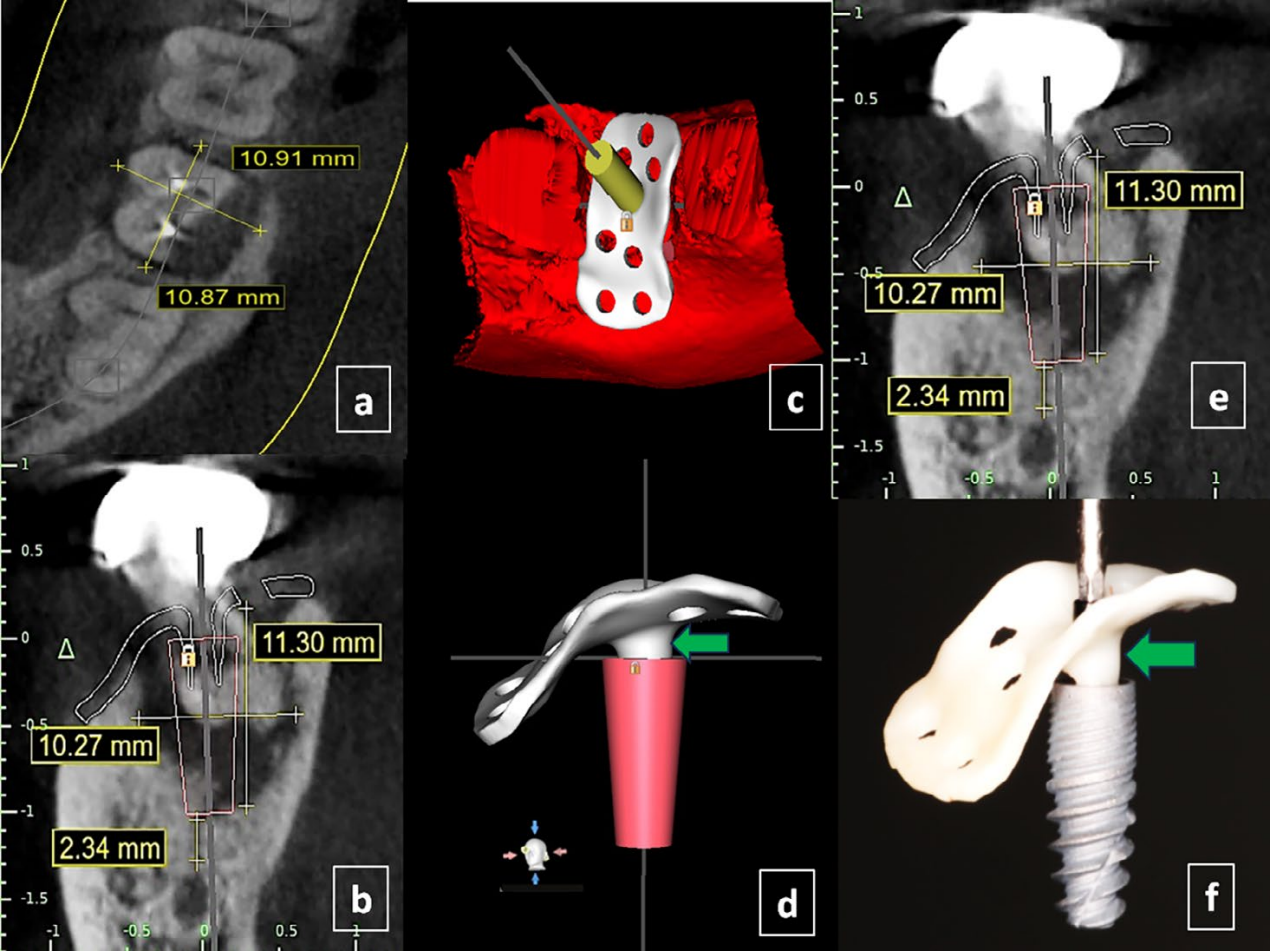
Digital planning of the implant and CZM in the mandible. (a, b) CBCT measures; (c) ZM in white color and axis of the future abutment in yellow; (d) connection of the implant (red) to the ZM connector at the inner surface (green arrow); (e) position of the assembly ZM/Implant; (f) ZM with its connector for clinical use.

Patients' DICOM (Digital Imaging and Communications in Medicine) data obtained from CBCT scan were transferred to the Bluesky Plan Software (Blueskybio, Illinois, USA) in order to virtually plan the precise position of the implant, the design of the CZM and the future prosthesis, as well. After using the segmentation function for segmenting the bone and teeth, artificial teeth, chosen from the software library, were placed on the edentulous span to simulate the appropriate position of the future prosthesis. The segmented bone and teeth, with the virtual implant (Naturactis Implant, Euroteknika, Lyra company, France), obtained from the software library, were exported as STLs file from the Bluesky Plan Software and then transferred to the Autodesk Meshmixer Software (Meshmixer, Autodesk Inc., USA). With the latter, maxillary bone augmentation, simulating the surgical sinus lift procedure, was performed using the sculpt tools, in order to obtain at least 1.5 mm of bone around the virtual implant. The Separate and Extrude functions were used for the design of the CZM. The CZM was created as a single continuous piece, and uniquely dimensioned to fit the zone of the bone defect after tooth extraction. The generated thickness was 1 mm. An extension was created at the inner surface of the CZM to serve as a connector or abutment used to connect the implant (hexagonal conical internal connection) to the CZM, secured with an abutment screw. Indeed, holes have been generated in the CZM in order to allow the vascularization of the surgical site and to stabilize the complex ZM/Implant using fixation screws (2 bone screws, 6 mm length, BTK's BT Screw kit, Italy) to the buccal and palatal/lingual cortical bone. The ZM was milled (Rolland DgShape 52di, Japan India) from a zirconia disc (BeautyZir Disk, 1200 Mpa, China) for clinical application (Figure 3a–h). Sintering, using a high‐temperature furnace (Zirkon Zahn, Italy), was performed at 1500°C. The zirconia meshes were cleaned, disinfected and sterilized at a temperature of 134°C following the steps previously described [8]. The same procedure of digital planning of the implant and CZM design and milling was performed in the mandible (Figure 4a–f). CAD/CAM of zirconia final crowns were also performed after digital impression and scanning.

**FIGURE 5:**
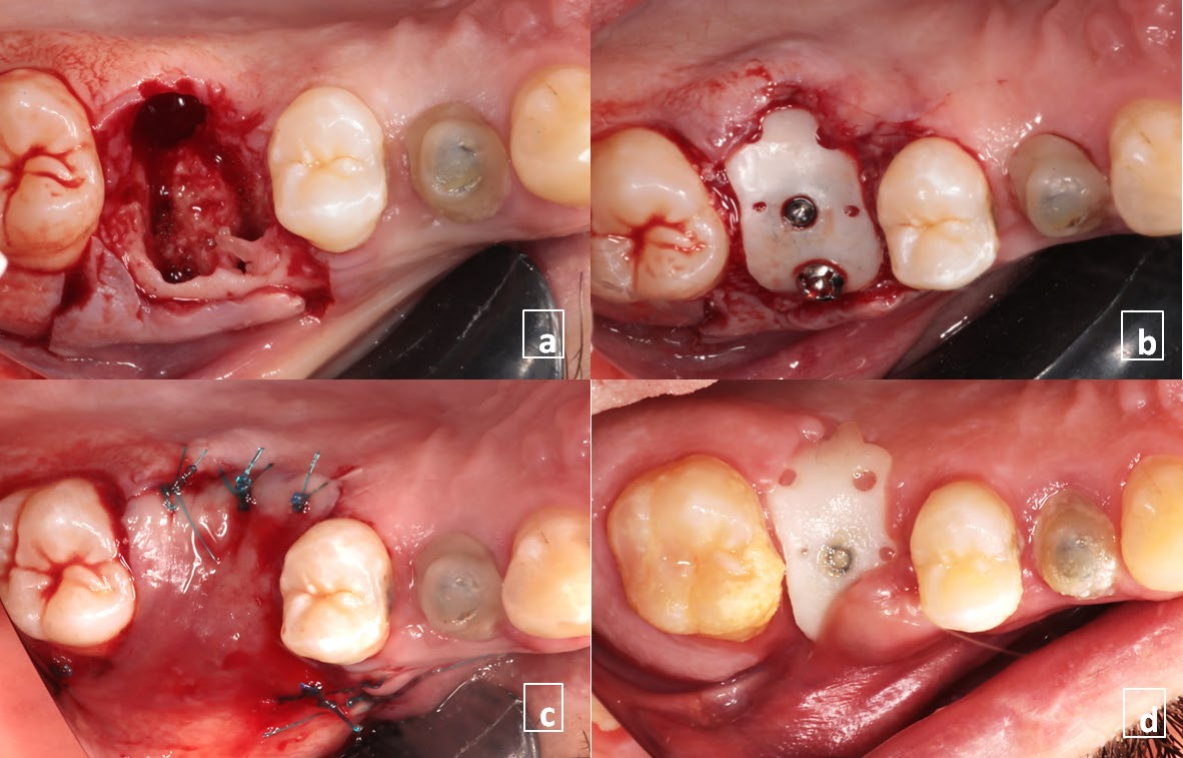
Clinical views; (a) extraction of the molar exposing the sinus membrane; (b) placement and screw fixation of the CZM/Implant assembly without osteotomy; (c) ZM recovery with a buccal flap; (d) ZM exposure.

Regarding the first patient, the initial treatment plan was the extraction of tooth #16 with socket preservation, and reassessment of the bone quality and quantity after four months for potential additional GBR before implant placement. However, as the patient requested a faster solution, simultaneous implant placement with a crestal sinus lift was proposed as an alternative approach. On surgery day, local anesthesia with a 4% solution of articaine and 1:100,000 epinephrine was performed. The remaining roots of the tooth were extracted, exposing the sinus membrane. A crestal lift of the sinus membrane, using surgical instruments from the Medesy Sinus lift Kit (Italy) was performed. The space created was then filled with bone graft material using bovine bone substitute mixed with sterile saline solution (OsteoBiol, Tecnoss Dental S.R.L, Italy), followed by the placement of the assembly CZM/Implant structure that was fixed to the cortical bone with screws through the holes in the membrane. The mesh was then covered by a buccal full‐thickness mucoperiosteal flap. Mucosal sutures were performed with Nylon sutures 4/0. After ten days, the patient reported no severe pain, though mild swelling was observed. The ZM began to be exposed but showed no signs of infection (Figure 5a–d).

**FIGURE 6:**
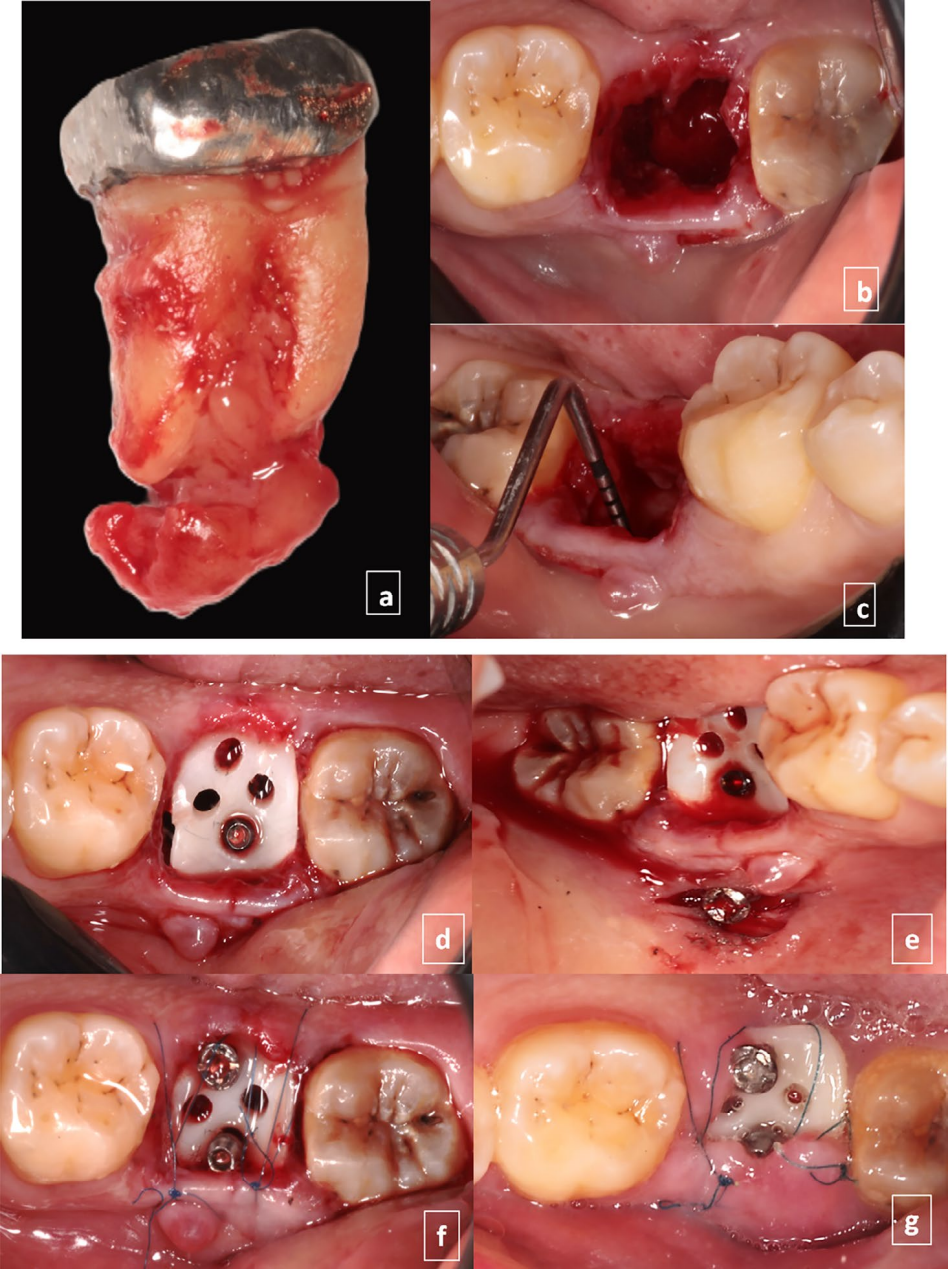
Clinical views; (a) extracted tooth with the appended periapical lesion; (b) fresh alveolar socket with no septa; (c) probing of the alveolar bone defect; (d) placement of the assembly ZM/Implant in the fresh socket with no osteotomy and screw fixation; (e) sutures; (f, g) exposure of the ZM.

The initial plan was to extract the tooth and place the implant connected to the CZM for stabilization, without the use of bone grafting. Under local anesthesia, with a 4% solution of articaine and 1:100,000 epinephrine, the tooth was extracted with the periapical lesion appended to apices and the buccal alveolar bone loss was assessed using a periodontal probe. The combined CZM/Implant structure was placed, with no osteotomy, and fixed with screws and sutured. No flap was used to cover the mesh (Figure 6a–g).

The two patients received a medical prescription including oral antibiotics (Amoxicillin 2 g/day for 6 days), injectable corticosteroids (8 mg/day for 3 days), and analgesics (Paracetamol, 2 g/day for 5 days). They were instructed to keep good oral hygiene and avoid brushing the surgical site.

## 5 Follow‐Up and Outcomes

**FIGURE 7:**
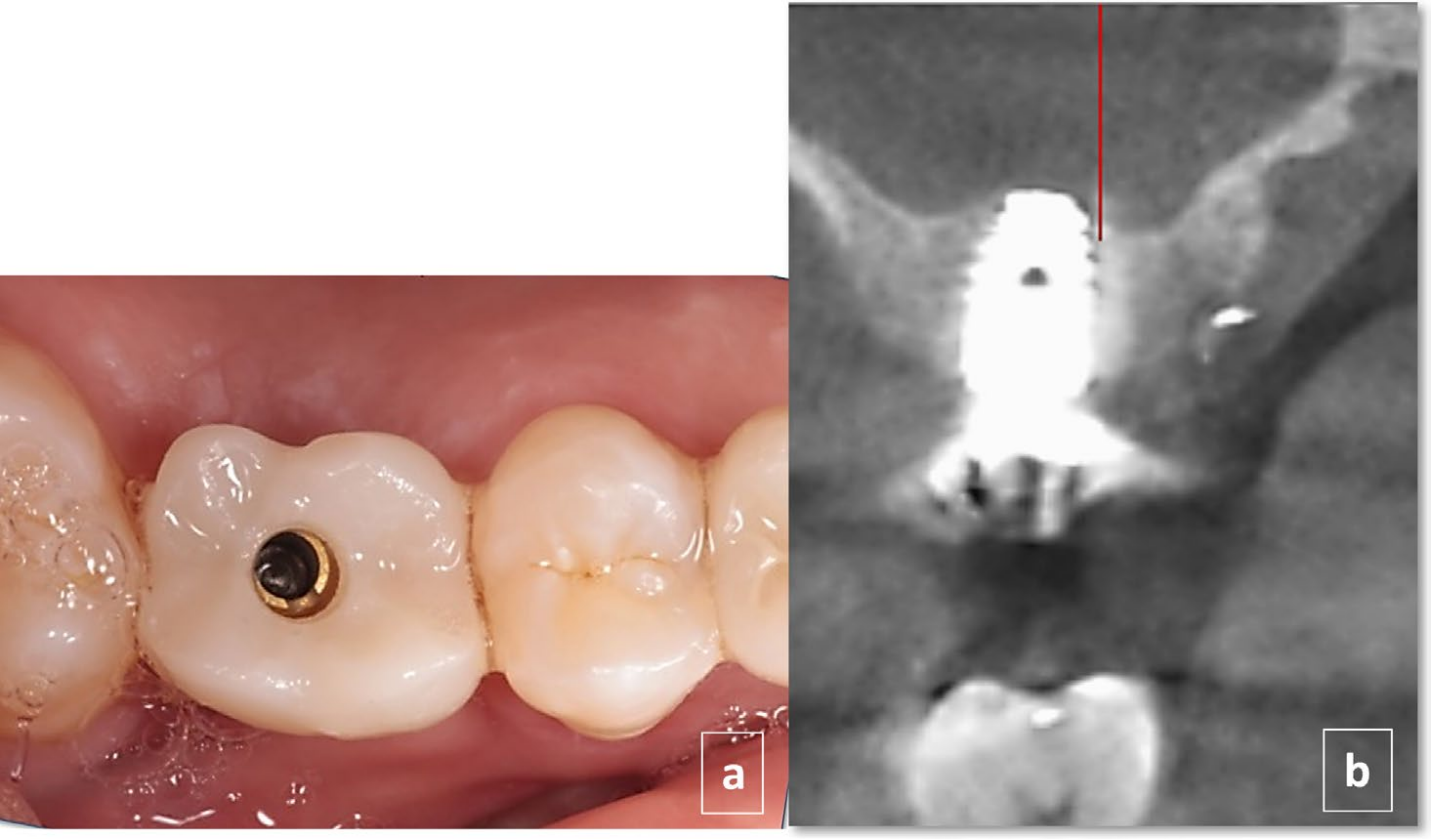
(a) Clinical view of the final zirconia crown; (b) CBCT coronal view showing bone growth with a follow‐up period of 2 years.

**FIGURE 8:**
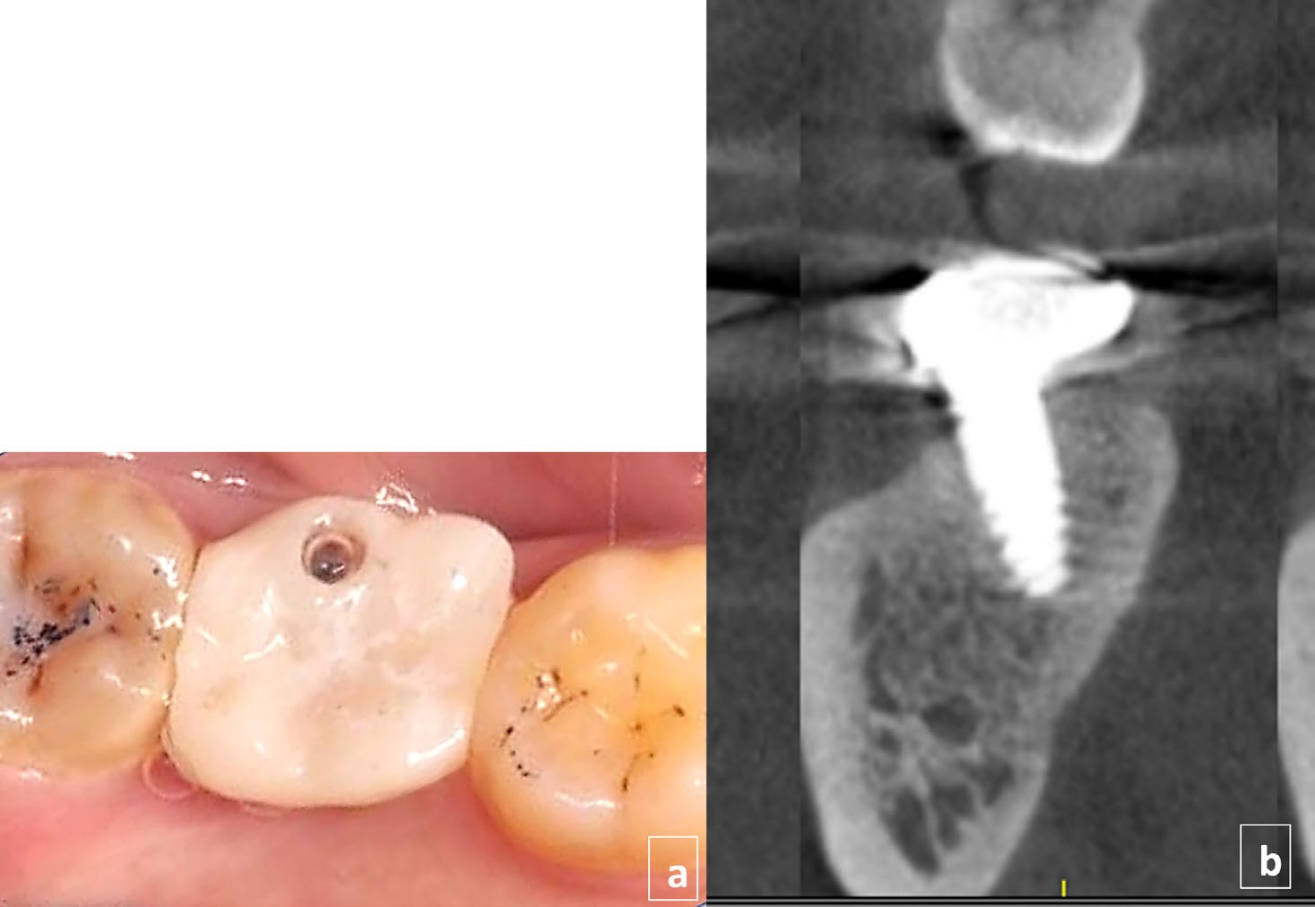
(a) Final zirconia crown; (b) CBCT coronal view of the bone healing after 2 years.

Healing was monitored through successive clinical controls. After 8 months, upon retrieval of the ZM, the implants were clinically stable. The CBCT scans showed significant bone growth confirming the good osteointegration. Healing abutments were placed, followed by the final zirconia crown on a titanium base. Upon loading, the patients experienced no pain, and the implant remained fully functional and exhibited, clinically, healthy peri‐implant tissues at the two‐year follow‐up (Figures 7a,b and 8a,b). Tables 1 and 2 provide a summary of patients' information and timeline of treatment steps, respectively.

**TABLE 1:** Summary of patients' information.

	Case 1	Case 2
Gender	Male	Female
Age (years)	30	36
Medical history	Healthy—No tobacco consumption	Healthy—No tobacco consumption
Reason for consultation	Infected roots of first maxillary right molar	Root fracture/chronic abscess (fistula) of second right mandibular molar
Clinical symptoms	No	Mild
Preoperative CBCT	Periapical lesion/bone loss	Large periapical lesion/significant bone loss

**TABLE 2:** Timeline of treatment steps.

Treatment steps	Procedures
Diagnosis	Oral examination: decayed tooth #16 and fractured tooth #47 IOS CBCT Measures: occlusal view #16: bone width mesio‐distal = 9.36 mm, bucco‐palatal = 13.32 mm #47: bone width mesio‐distal = 10.91 mm, bucco‐palatal = 10.87 mm Coronal view #16: bone height = 2.02 mm #47: distance between the implant apex and MAN = 2.34 mm
Digital workflow	Data acquisition and integration Position of the implant in the axis of the future prosthesis Implant diameter/length: 40/10 mm 1st and 2nd case Connection of the implant to a connector designed in the CZM (CAD) Pores in the CZM for vascularization and pores for screw fixation to the buccal and palatal/lingual walls Milling of CZM (CAM), sintering and finishing
Surgery	Teeth extraction (#16 and #47) Sinus lift elevation for the 1st case using bovine bone graft Immediate implantation using the CZM/Implant No osteotomy Full‐thickness mucoperiosteal flap in the 1st case
Drug prescription/oral hygiene instructions	Antibiotics, corticosteroids, analgesics Mouthwash: chlorhexidine 0.12%
Prosthetic Restoration CAD/CAM	8 months after‐Healing phase and osseointegration Regular scan bodies IOS CAD/CAM Fbrication of Zirconia crowns, sintering and finishing Screw fixation and composite resin filling of the access hole
Follow‐up	48 months
Outcome	Favorable with no crestal bone loss. No complications

Abbreviations: CAD, Computer‐Aided Design; CAM, Computer‐Aided Manufacturing; CBCT, Cone Beam Computed Tomography; CZM, Customized Zirconia Membrane; IOS, Intraoral Oral Scan.

## 6 Discussion

In these clinical case reports, we presented a new technique for ensuring the stability of the implant during immediate implantation, in compromised sockets, without osteotomy or drill, using an implant connected through a prosthetic screw to a connector created at the inner surface of a fitted CZM. The perforated CZM covering the fresh sockets was attached through screws to the buccal and palatal/lingual cortical bone, thus preventing micromovements that could compromise osseointegration and may lead to implant failure. In these specific cases, we do not refer to primary stability, since there has been no drilling into the bone to stabilize the implant. To the best of our knowledge, this is the first description of an innovative approach called “the hanged technique,” in which the implant, suspended within the fresh extraction socket, is stabilized by its connection to the CZM.

Digital technology has revolutionized dentistry. The integration of intraoral data and radiographic data through virtual planning software allows a precise 3‐dimensional visualization of the position of the implant and the ZM, predicting the future position of the prosthetic restoration [9, 10]. Another advantage is that the design of ZM is specific to each patient, resulting in an immediate fit to the alveolar socket and a significant reduction in intraoperative time [11]. The use of CZM is recent and reported as case reports and case series [12, 13]. However, despite these advantages, the production of CZM requires sophisticated digital planning and milling techniques, which might not be readily available in every clinical environment. Additionally, there is a shortage of long‐term clinical data regarding the effectiveness of ZMs in immediate implant placement, highlighting the need for more research to confirm their reliability and durability. Moreover, the rigid nature of zirconia may limit its adaptability to complex anatomical structures, potentially reducing its effectiveness in highly irregular defects.

It should be noted that patients are young and healthy. The first case involved the first upper right molar with periapical lesions and bone loss, and the second case involved the second mandibular molar with a large periapical lesion and a significant amount of bone loss. The advantages of immediate implant placements, compared to delayed methods, are well documented in the literature. The placement of the implant into the socket after tooth extraction allows the preservation of the alveolar bone and the significant reduction of bone resorption [14]. Indeed, the time period needed for the bone healing and the surgical procedure for the placement of the implant are removed. Although this technique improved patients' satisfaction, correct selection of cases, taking patients' related factors into consideration, and accurate indication for immediate implant placement is crucial for a more predictable outcome and a long‐term success [15].

The objective of the use of the graft in the first case was for crestal sinus augmentation in a patient with residual bone < 3 mm [16]. The space created after sinus membrane elevation was filled using bovine xenograft, an alternative bone substitute [17]. Immediate implantation in case 2 was performed without the use of additional bone substitutes, despite the significant amount of bone resorption induced by the periapical lesion. Moreover, we didn't use a flap in the second case, since it doesn't prevent membrane exposure. We insisted that patients take care not to stress mechanically the membrane, considering that the risk of infection is reduced, even if the membrane is exposed due to resistance to bacterial infection. Indeed, improved soft tissue integration is achieved using ZM [18]. It's worth noting that in these cases presenting with infected teeth, no symptoms of infection were reported after teeth extraction and immediate implant placement. The patients received a medical prescription including antibiotics [19], injectable corticosteroids and analgesics. They were instructed to keep a good oral hygiene using a mouthwash and to avoid brushing the surgical site. In recent years, high success rates of immediate implant placement have been reported in sockets with periapical diseases after adequate removal of the lesion [20, 21]. Finally, the outcome was favorable with a follow‐up period of 2 years. Patients were very satisfied in terms of time saving, less surgical intervention, absence of complications and very good outcome with fully functional zirconia prosthesis. Tables 1 and 2 (timeline) represent a summary of patients' information, intervention and outcome.

## 7 Limitations

The present new concept has included only two clinical cases. A comparative study, taking into account strict inclusion and exclusion criteria, will be conducted as part of a research project to validate the effectiveness of the technique. Indeed, ISQ values should have been assessed to confirm secondary implant stability and osseointegration, thereby improving the time between immediate implant placement and the final prosthesis.

## 8 Conclusion

Based on digital technology, we do think that the new Hanged Concept is promising, potentially reducing mechanical contraindications for immediate implants in compromised sockets. It provides clinicians with greater control over implant axis in sockets with minimal bone, requiring fewer steps for patients compared to other methods. A research clinical study including a large sample of patients is planned to assess clinical outcomes and validate this new concept.

## Author Contributions

**Laith Glissa:** conceptualization, formal analysis, investigation, methodology, project administration, software, validation, visualization, writing – original draft, writing – review and editing. **Latifa Berrezouga:** data curation, formal analysis, investigation, project administration, resources, supervision, validation, writing – original draft, writing – review and editing.

## Funding

The authors have nothing to report.

## Ethics Statement

The clinical project was submitted by the authors (L.G. and L.B.), according to the Declaration of Helsinki involving human subjects, to the Ethics Committee of the faculty of medicine of Monastir, Tunisia; and was approved under the following reference number IORG 0009738 N°198 OMB 0990‐0279.

## Consent

Written consent was obtained from all patients.

## Conflicts of Interest

The authors declare no conflicts of interest.

## Data Availability

The data used in the present study are available from the corresponding author upon reasonable request.
